# Gut microbiome markers in subgroups of HLA class II genotyped infants signal future celiac disease in the general population: ABIS study

**DOI:** 10.3389/fcimb.2022.920735

**Published:** 2022-07-25

**Authors:** Patricia L. Milletich, Angelica P. Ahrens, Jordan T. Russell, Joseph R. Petrone, Meghan A. Berryman, Daniel Agardh, Jonas F. Ludvigsson, Eric W. Triplett, Johnny Ludvigsson

**Affiliations:** ^1^ Department of Microbiology and Cell Science, Institute of Food and Agricultural Sciences, University of Florida, Gainesville, FL, United States; ^2^ Department of Clinical Sciences, Lund University, Malmö, Sweden; ^3^ Department of Medical Epidemiology and Biostatistics, Karolinska Institute, Stockholm, Sweden; ^4^ Department of Pediatrics, Örebro University Hospital, Örebro, Sweden; ^5^ Crown Princess Victoria’s Children’s Hospital and Division of Pediatrics, Department of Biomedical and Clinical Sciences, Linköping University, Linköping, Sweden

**Keywords:** celiac disease, autoimmunity, human leucocyte antigen, infant, gut microbiome

## Abstract

Although gut microbiome dysbiosis has been illustrated in celiac disease (CD), there are disagreements about what constitutes these microbial signatures and the timeline by which they precede diagnosis is largely unknown. The study of high-genetic-risk patients or those already with CD limits our knowledge of dysbiosis that may occur early in life in a generalized population. To explore early gut microbial imbalances correlated with future celiac disease (fCD), we analyzed the stool of 1478 infants aged one year, 26 of whom later acquired CD, with a mean age of diagnosis of 10.96 ± 5.6 years. With a novel iterative control-matching algorithm using the prospective general population cohort, All Babies In Southeast Sweden, we found nine core microbes with prevalence differences and seven differentially abundant bacteria between fCD infants and controls. The differences were validated using 100 separate, iterative permutations of matched controls, which suggests the bacterial signatures are significant in fCD even when accounting for the inherent variability in a general population. This work is the first to our knowledge to demonstrate that gut microbial differences in prevalence and abundance exist in infants aged one year up to 19 years before a diagnosis of CD in a general population.

## Introduction

Celiac disease (CD) is an antigen-driven, immune-mediated disorder occurring in 1% of the Western population and increasing in incidence (Lebwohl et al., 2015; Sharma et al., 2019; King et al., 2020). The presence of gluten drives intestinal inflammation and the production of autoantibodies against tissue transglutaminase (tTG) in genetically predisposed individuals (Sollid and Lie, 2005).

Gliadin, a prolamin found in gluten, is rich in glutamine and proline, making it partially resistant to gastrointestinal digestion (Shan et al., 2002). When glutamine-rich gluten peptides reach the lumen, they interact with tTG, deaminating glutamine into glutamate. This deamination causes the peptide to become negatively charged and preferentially binds to the class II human leukocyte antigen (HLA) molecules DQ2 and DQ8 for the presentation to CD4+ T cells (Sollid, 2017). HLA proteins encode the human equivalent of the major histocompatibility complex (MHC) proteins, which display antigens for T-helper cell recognition to elicit tolerance or immune response. Protein structure altering allelic polymorphisms found in DQ2.5, DQ2.2, or DQ8 form regions P4 and P6 for DQ2.5 and DQ2.2, and P1 and P9 for DQ8 that attract negatively charged residues of glutamate making an individual more susceptible to celiac (Moustakas et al., 2000; Tollefsen et al., 2006). HLA is strongly associated with CD, with DQ2/DQ8 found in up to 98% of CD patients (Cecilio and Bonatto, 2015).

It is estimated that approximately 30% of the world population carries either DQ2 or DQ8. Yet only 1% of those with the risk DQs haplotypes are diagnosed with CD, pointing toward other factors that increase the risk for or protect from the disease (Mustalahti et al., 2010; Megiorni and Pizzuti, 2012; Jamnik et al., 2017). Research suggests that intestinal dysbiosis is characteristic of CD etiology, with aberrant microbiome signatures presenting even in individuals who have followed a gluten-free diet (Collado et al., 2009; Wu et al., 2021).

Prospective studies of gut dysbiosis in CD are limited, especially in the years preceding a diagnosis. Environmental and genetic mediators of the infant gut microbiome have been previously described, including mode of delivery, infant feeding (e.g., duration of breastfeeding), gestational age, antibiotic (Penders et al., 2006), and, recently, HLA genetics (Russell et al., 2019). The influence of HLA-DQ and environmental factors on gut colonization continues to be studied. However, these studies focus primarily on infants already at high risk for CD (Palma et al., 2012; Olivares et al., 2015). Restricting the study of gut microbiota to infants with a first-degree relative with CD may lead to conclusions that are not generalizable to the larger population, and cross-sectional study designs limit the identification of early microbial risk marks for future celiac disease (fCD).

The current investigation was derived from the general population cohort, All Babies In Southeast Sweden (ABIS), which sought to identify early risk and protective factors for future disease. Here, ABIS allowed for the study of the contribution of HLA genetics and environmental factors to increased CD risk and for the discovery of microbial differences in infants at one year of age who developed CD up to two decades later.

## Materials and methods

### Study population

This investigation derives from the ABIS prospective, population-based cohort of 17,055 children born in southeast Sweden from October 1, 1997, to October 1, 1999. The ABIS cohort was designed to identify environmental and genetic factors associated with autoimmune diseases (Ludvigsson et al., 2001). After being provided oral, written, or video information, parents gave their informed consent for their child’s participation. Mothers completed questionnaires and diaries for the first year of life, which included but were not limited to information on gestational age, mode of delivery, antibiotic use, age of introduction or frequency of consumption of certain foods (fruit, vegetables, eggs, meat, gluten, among others), duration of breastfeeding, and environmental factors and toxic exposures. Infants were binned into geographical regions based on their municipality at one year of age to help account for cultural differences possibly contributing to the microbiome structure (**Figure S1**).

### Sample collection

Stool samples were collected from infants’ diapers using provided sterile spatula and tubes. These samples were immediately frozen after collection. Samples collected at home or at the WellBaby Clinic were sent to the Research Laboratory of Division of Pediatrics, Medical Faculty, Linkoping University, where all samples were stored at -80°C. Samples for this study were shipped to the University of Florida in a frozen state and stored at -80°C until extraction (Russell et al., 2019).

### Institutional review board approval

The ABIS study was approved by the Research Ethics Committees of the Faculty of Health Science at Linköping University, Sweden, 1997/96287 and 2003/03-092 and the Medical Faculty of Lund University, Sweden (Dnr 99227, Dnr 99321) as described previously (Russell et al., 2019). The microbiome analysis performed at the University of Florida was approved by the University of Florida’s Institutional Review Board as an exempt study IRB201800903.

### Diagnoses from the swedish national patient registry

The Swedish Patient Register provided data on autoimmune and neurological disorders in ABIS children, with the most recent update having occurred in 2021 (international classification of disease, ICD-10: K90) (Ludvigsson et al., 2011). For a diagnosis of CD for this investigation, at least two records of CD in the Patient Register were necessary. Up until 2012, Swedish pediatricians required both positive serology and a positive small intestinal biopsy for diagnosis. A validation study found that 67/67 (100%; 95%CI=95–100%) of Swedish pediatric gastroenterologists performed a small intestinal biopsy in >90% of all children with suspected CD prior to diagnosis (Ludvigsson et al., 2011). That same validation study found that in a Swedish setting, 95% of individuals with Marsh III (villous atrophy) had CD (Ludvigsson et al., 2011). In 2012, ESPGHAN offered a non-biopsy pathway in selected children with levels of tTG antibodies ten times the upper limit of normal, and these recommendations were re-iterated by the Swedish working group for pediatric CD (Husby et al., 2012).

### HLA genotyping

HLA genotyping was performed with the available blood spots of the ABIS children. HLA-DR-DQ genotypes are defined by the presence of common European HLA-DR-DQ alleles associated with disease risk using sequence-specific hybridization with lanthanide-labeled oligonucleotide probes (Ilonen et al., 2016).

The CD-associated HLA genotypes were categorized as a combination of known risk alleles, HLA-DQA1*05:01-DQB1*02 (DQ2.5), DQA1*03:01-DQB1*03:02 (DQ8), DQA1*02:01-DQB1*02 (DQ2.2), possibly protective HLA-DRB1*01:01/DQA1*01:01-DQB1*05:01 (DR1-DQ5), and CD non-associated HLA-DQs (DQX).

### Generation of amplicons for microbiome analysis

Microbial composition analysis was run in stool samples collected at one year of age. DNA extraction, 16S rRNA barcoded PCR, and V3-V4 16S rRNA Illumina sequencing were performed as previously described (Russell et al., 2019). Bacterial quantification through universal 16s rRNA primers was performed as described previously (Russell et al., 2021).

Forward and Reverse fastq reads were merged using default PEAR parameters, and ambiguous primers were removed using Cutadapt using the following parameters: Forward, NNNNCCTACGGGAGGCAGCAG -e 9. Reverse, ATTAGATACCCSBGTAGTCCCC -e 11 (Zhang et al., 2014; Martin, 2011). ASVs were left untruncated and set to a minimum length of 400 nucleotides before filtering using the DADA2 R package (Callahan et al., 2016; R Core Team, 2016). Sequences were filtered to allow no ambiguous nucleotides, a maximum expected error rate of 2, Q-score 10, and PhiX reads removed. Sequences were then “pseudo-pooled” before the chimeras were removed with the default consensus method. Taxonomy was assigned using the SILVA v138 database (Quast et al., 2013).

### Population subset and filtering

Infants with fewer than 1000 raw reads were not included in this analysis, nor were those later diagnosed with a neurological disorder or an autoimmune disease other than CD, resulting in 1452 controls and 26 fCD. Other excluded autoimmune diseases include hypothyroidism, type-1 diabetes, juvenile idiopathic arthritis, Crohn’s disease, or ulcerative colitis.

The average read counts per sample were 46627, with a maximum of 632001. In this analysis, 10389 ASVs were filtered to only include those present in at least five infants at five or more reads, resulting in 2376 ASVs that were then binned to 184 unique genera. Relative abundances were calculated using the transform_sample_counts function, and ASVs belonging to the same genus were combined using tax_glom (McMurdie and Holmes, 2013). To calculate total abundance, the relative abundance values were multiplied by sample by the total number of copies of 16s rRNA per gram of stool, as determined through qPCR. Significance p-values in the microbiome analysis were adjusted for false discovery rate (FDR). The statistical program R Foundation for Statistical Computing was used for all computational analysis (R Core Team, 2018).

### Early life factors associated with fCD

To determine factors associated with fCD, chi-square tests were conducted to compare the year-one diary responses (n=1478) using Monte Carlo simulation.

### Confounders of beta microbial diversity

Confounders of beta diversity were assessed within the total population (n = 1478) at the genus level. Subjects with missing data for each variable were excluded from the calculation. The distance function from the phyloseq package was applied using the binomial method. The distance matrix was tested against the variable of interest using default parameters with the adonis2 R package (Oksanen et al., 2020).

### Random permutations for matched case-control iterations

**Figure 1:**
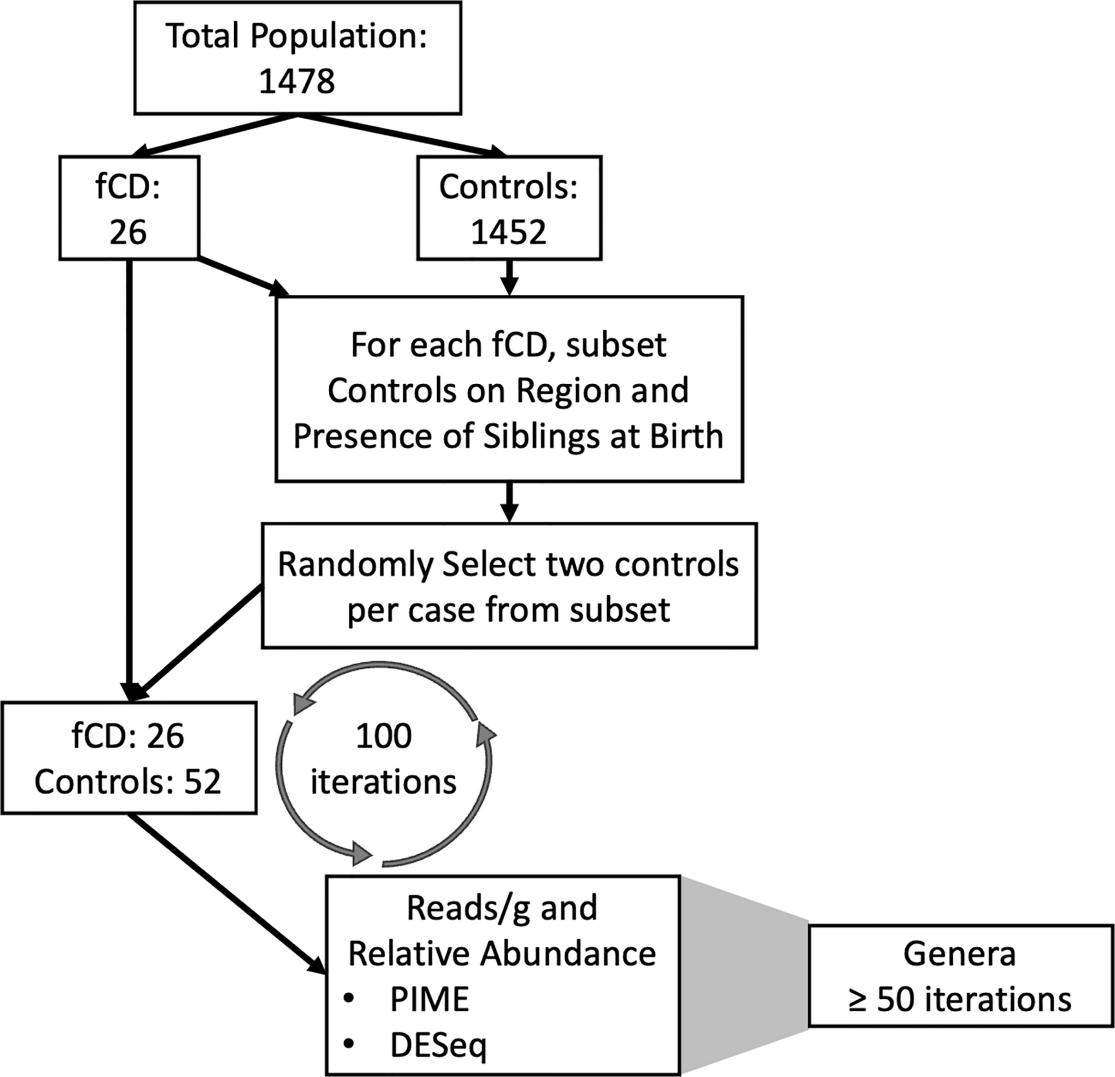
Pipeline for the matching iterations algorithm and analysis of future celiac disease (fCD). The differential prevalence (The Prevalence Interval for Microbiome Evaluation, PIME) and abundance (DESeq2) analyses were conducted independently at each for 100 iterations of matched controls with the fCD cases. Matches were randomly selected from subsets based on microbiome confounders of geographic region and siblings at birth. The random selection of controls was applied to investigate bacterial differences in cases and controls while accounting for inherent variability within controls in the general population. Genera that were of most interest to this investigation were defined as those that were shared across at least fifty iterations of randomized case-control groups.

To compensate for the 55-fold increase of controls to fCD infants, 100 random selections of controls were employed to create 100 separate randomized matched controls for our comparisons (**Figure 1**). The iterative process allowed us to account for the inherent variability within the controls in a general population cohort without the risk of overpowering the results with an imbalance of controls. For each iteration, controls were selected for each future celiac disease infant (fCD) to balance geography (region of Sweden) and the presence of siblings at birth in the comparative groups. These variables were selected because they were identified as confounders of beta diversity in this cohort (ANOVA: padj < 0.05) (**Table S1**). From the resulting total subset of controls, two infants were randomly selected as matches for each fCD infant. This process was performed 100 times. The set.seed() function was used for each iteration to allow for replication of case-control permutation groups.

### Differentially abundant genera in fCD versus controls

Differential abundances of microbes without a prevalence threshold were assessed with the binomial distribution model R package, DESeq2 (Love et al., 2014). For both the total abundance and relative abundance analyses, the estimateSizeFactor() function was used with “poscount” type, allowing for zeroes. Within DESeq2, the Wald test for calculating p values, with a local fit type, was employed. The additional Cook’s distance filtering was not considered. After each iteration of DESeq2 (within each of the 100 case-control permutations), significance values were adjusted for multiple comparisons across the genera. Genera were deemed significant to this investigation if they appeared in at least 50 of the 100 iterations of case-control matching with a padj < 0.05.

### Prevalence filtering of the core microbiome in fCD

Prevalence is a metric that is often overlooked in microbiome studies. Prevalence is defined as a proportion of samples in a specific group that share taxa, irrespective of abundance. The Prevalence Interval for Microbiome Evaluation (PIME) was employed at each of the 100 case-control group comparisons to obtain the core genera representing fCD or controls and to determine whether any bacteria were more likely to be present or absent in fCD (Roesch et al., 2020). The PIME package was designed as a robust filtering methodology for low prevalence, and its reliability has been validated against existing methods with several 16S rRNA independent datasets. PIME determines the set of bacteria that meet the prevalence criteria at increasing increments of 5%, and the optimal prevalence threshold is selected by the user, balancing out of bag error (OOB) rates while maximizing the representation of the bacteria (i.e., number of taxa retained after filtering).

The first ten iterations of case-control comparisons were used as guides for the expected OOB in the remaining ninety iterations. The initial OOB for the first ten runs ranged from 33.3% to 42.3%, with an average of 38.0%. An optimal prevalence threshold of 60% was selected for these analyses, as an average OOB error of 2.82 was observed at this interval, with an average of 35 of the original 184 genera (19%) and 5.02E9 of the original sequences retained. After filtering for prevalence, binomial distances for iterations 25, 50, 75, and 100 were conducted using plot_ordination and stat_ellipse (Wickham, 2016).

To determine which genera consistently differentiated controls and fCD using this approach, the top 30 taxa with the highest mean decrease accuracy (MDA) were considered at each iteration. Taxa were removed with negative MDA values, and the mean abundance for the remaining taxa were determined and binned for all iterations. Taxa were removed if they were significant in fewer than 50 of the 100 iterations. The direction of the effect was assessed for each remaining genus with respect to the average abundance in fCD and controls.

### Conditional factors

Kruskal-Wallis or Mann-Whitney U tests were used to determine the effect of environmental and genetic factors against the total abundance and relative abundance of the significant genera identified in the DESeq2 or PIME analyses. These tests were conducted at the level of the complete cohort (n=1478). P-values were adjusted using false-discovery-rate. Factors that were significant in both total abundance and relative abundance are presented in **Table S3**.

## Results

### Diagnoses

Twenty-six subjects were diagnosed with CD according to the Swedish National Patient Registry (Walker-Smith and Sanderson, 1999). Female subjects (n=17, 65%) had a mean age of diagnosis of 10.71 ± 5.17 years, while the male age of diagnosis was 11.44 ± 6.65 years (**Table 1**).

**Table 1:** Distribution of genetic and environmental factors for infants with future CD.

fCD	Age of Diag.^A^	Human Leukocyte Antigen (HLA)Genotype	Sex	Mode of Delivery	Region	Siblings at Birth	Total BF^B^	Gluten Intro.^C^	Control Subset (N)
1	2	NA	Male	NA	North	No	1 to 3	4 to 7	177
2	3	DR3-DQ2.5/DR4-DQ8	Female	Cesarean	North	Yes	1 to 3	NA	230
3	3	DR3-DQ2.5/DR15-DQ602	Female	Vaginal	East	Yes	8 to 9	4 to 7	143
4	4	DR4-DQ8/DR7-DQ2.2	Male	Vaginal	East	Yes	4 to 7	4 to 7	143
5	4	DR4-DQ8/DR7-DQ2.2	Female	Cesarean	South	Yes	8 to 9	4 to 7	199
6	4	DR3-DQ2.5/DR5-DQ7	Female	Vaginal	East	Yes	4 to 7	4 to 7	143
7	5	DR3-DQ2.5/DR3-DQ2.5	Female	Vaginal	West	Yes	8 to 9	8 to 9	242
8	6	DR4-DQ8	Male	Vaginal	North	Yes	8 to 9	4 to 7	230
9	8	DR4-DQ8/DR4-DQ8	Female	Vaginal	West	No	8 to 9	8 to 9	156
10	9	DR3-DQ2.5/DR13-DQ604	Male	NA	North	NA	8 to 9	4 to 7	426
11	10	DR3-DQ2.5/DR9-DQ9	Female	Vaginal	South	Yes	1 to 3	4 to 7	199
12	11	DR3-DQ2	Male	Vaginal	West	Yes	1 to 3	4 to 7	242
13	12	DR5-DQ7/DR7-DQ2.5	Female	Vaginal	North	No	4 to 7	8 to 9	177
14	13	DR3-DQ2.5/DR15-DQ602	Female	Vaginal	South	Yes	1 to 3	4 to 7	199
15	13	DR4-DQ8/DR5-DQ7	Female	Vaginal	South	Yes	1 to 3	4 to 7	199
16	13	DR3-DQ2.5/DR15-DQ602	Female	Vaginal	East	No	4 to 7	8 to 9	95
17	14	NA	Female	Vaginal	East	Yes	8 to 9	4 to 7	143
18	14	DR3-DQ2.5/DR4-DQ8	Female	Vaginal	East	No	1 to 3	4 to 7	95
19	15	DR4-DQ8/DR15-DQ602	Male	Vaginal	South	Yes	8 to 9	4 to 7	199
20	15	DR4-DQ7/DR13-DQ603	Female	NA	North	No	8 to 9	4 to 7	177
21	16	DR3-DQ2.5/DR4-DQ8	Female	Vaginal	North	Yes	8 to 9	4 to 7	230
22	17	DR3-DQ2.5/DR8-DQ4	Male	Vaginal	North	No	8 to 9	8 to 9	177
23	17	DR3-DQ2.5/DR15-DQ602	Female	Vaginal	East	Yes	8 to 9	4 to 7	143
24	18	DR4-DQ8/DR13-DQ604	Female	Vaginal	West	Yes	8 to 9	4 to 7	242
25	19	DR4-DQ8/DR15-DQ602	Male	Vaginal	West	Yes	NA	NA	242
26	20	DR3-DQ2.5/DR7-DQ2.2	Male	Vaginal	North	Yes	8 to 9	4 to 7	230

Controls were selected by matching geographical region and presence of siblings at birth to each case.

^A^Age of celiac disease diagnosis.

^B^Duration of total breastfeeding.

^C^Month of gluten introduction.

### Core microbiome signatures: Bacteria with differential prevalence in fCD

**Figure 2:**
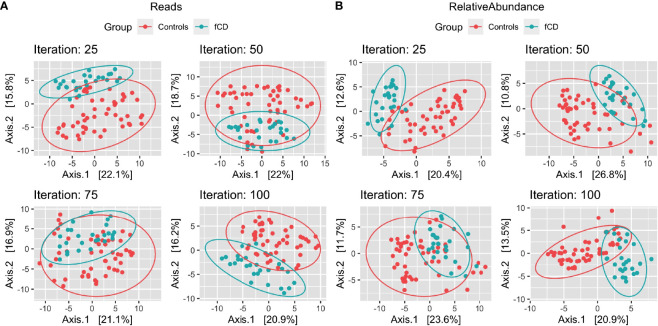
Microbiome diversity differences in selected case-control iterations for future celiac disease (fCD). Principal component analysis (PCoA) of the binomial distance of genera between fCD (n=26) and controls (n=52) is presented for Iteration #25, Iteration #50, Iteration #75, and Iteration #100, depicting the unique clustering of the core microbiome after applying a prevalence filter at 60% in PIME. Data are presented for **(A)** reads/g (total abundance) and **(B)** relative abundance. The percentages of the variance explained by each of the two components are presented in the axes.

With a prevalence threshold of 60% (**Table S2**), distinct clustering of fCD infants (n=26) and controls (n=52) was observed (**Figure 2** ).

**Figure 3:**
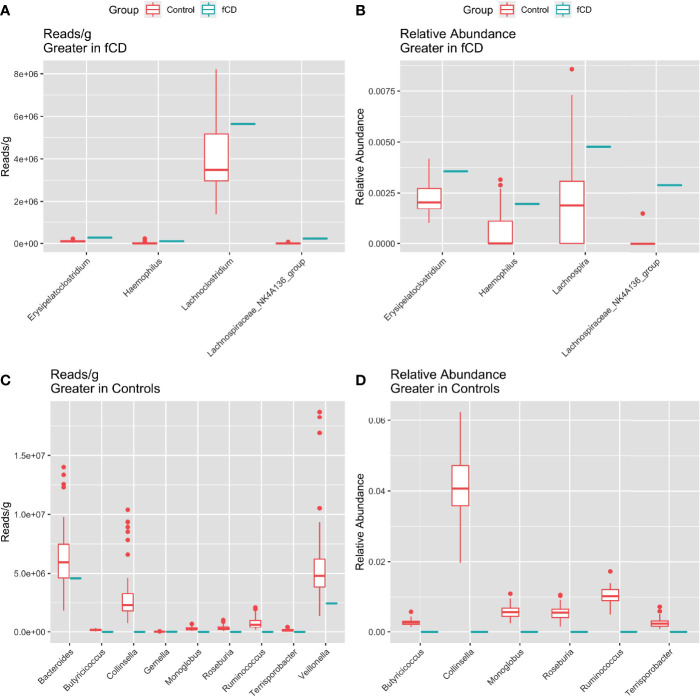
Distribution of the total (reads/g) and relative (%) abundance of genera differentially abundant in future celiac Disease (fCD) or controls. **A, C)** relative abundance **B, D)** core bacteria split by trends of abundance. **(A, B)** Bacteria greater in fCD infants. **(C, D)** Bacteria greater in controls.

Five genera were higher in fCD infants than controls, including three that were shared in both read/g (total abundance) and relative abundance analyses: *Erysipelatoclostridium*, *Haemophilus*, and *Lachnospiraceae NK4A136 group*. *Lachnoclostridium* only appeared in the reads/g analysis, while *Lachnospira* was only present in the relative abundance analysis (**Figures 3A**, **B** **)**.

Alternatively, nine genera were found higher in controls than fCD infants, six of which were found in both types of abundance analysis, including *Butyriococcus*, *Collinsella*, *Monoglobus*, *Roseburia*, *Ruminococcus*, and *Terrisporobacter* (**Figures 3C**, **D**). Three other genera, *Bacteroides*, *Gemella*, and *Veillonella*, were only present in the reads/g analysis.

### Differentially abundant microbes in fCD and controls

**Figure 4:**
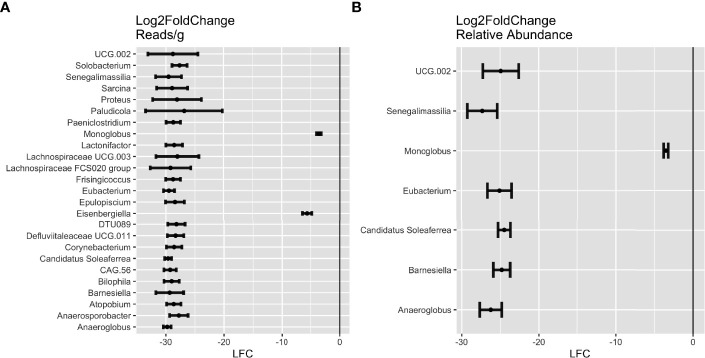
Differential abundance of genera in future celiac disease (fCD). DESeq Log2FoldChange (LFC) statistics of genera found in at least half of the matching iterations of cases and controls in **(A)** reads/g (total abundance) and **(B)** relative abundance. Genera with LFC < 0 are more abundant in controls than in fCD.

Across the iterations of matched case-control, 25 genera were consistently in higher abundance in controls (DESeq: p-adj ≤ 0.05), seven of which were found in both reads/g (total abundance) and relative abundance analyses. These include *Anaeroglobus*, *Barnesiella*, *Candidatus Soleaferrea*, *Eubacterium*, *Monoglobus*, *Senegalimassilia*, and *Oscillospiraceae UCG.002* (**Figure 4**)*. Candidatus Soleaferrea* was found to be completely absent in fCD infants but was present in an average of 21.7% of controls. The remaining 18 genera were found only in the reads/g analysis, five of which were also completely absent in controls (**Figure S2**).

### Genetic and environmental impact on bacteria

To determine potential causes of the taxonomic differences between fCD and controls, environmental and genetic factors were tested against the abovementioned bacteria that were shared in both relative abundance and read/g (total abundance) of the core microbiome and differential expression analyses (**Table 2** and **Table S3**).

**Table 2:** Factors associated with prevalence or abundance of gut bacteria enriched or depleted in fCD.

		HLA	Diet	Immunological	Other
PIME	*Butyricicoccus*	-DR13-DQ603 -DR15-DQ602			Residence Type
	*Collinsella*	-DR13-DQ603	-Weekly meals with Eggs^B^ -Duration of Exclusive Breastfeeding^A^ -Month of formula Introduction^A^		-Parents Abroad^C^
	*Erysipelatoclostridium*	-DR14-DQ5		-Pain Killer Medications^C^	
	*Lachnospiraceae* *NK4A136 group*	-DR15-DQ602		-Gastroenteritis^D^	-Sex -Residence Type
	*Haemophilus*				
	*Roseburia*			-Gastroenteritis^D^	
	*Ruminococcus*			-Otitis^D^ -Gastroenteritis^D^	-Sex -Residence Type
	*Terrisporobacter*	-DR14-DQ503			
DESeq2	*Anaeroglobus*		-Duration of Exclusive Breastfeeding^A^ -Duration of Total Breastfeeding^A^		-Mother Over 35
	*Barnesiella*	-DR7-DQ2.2			
	*Candidatus* *Soleaferrea*			-Infection^C^	-Sex -Smoking^C^
	*Eubacterium*		-Weekly meals with Eggs^B^	-High Blood Pressure Meds^C^	-Sex -Residence Type
	*Senegalimassilia*		-Duration of Total Breastfeeding^A^	-Antibiotics^C^ -Infection^C^ -Otitis^D^	-Sex -Parents Abroad^C^ -Worry Chronic Illness Child^C^ -Smoking^C^ -Risky Alcohol^C^
	*UCG.002*		-Weekly meals with Beef^B^		-Risk acts during pregnancy
Both	*Monoglobus*	-DR13-DQ603 -DR3-DQ2.5			-Sex

Environmental and genetic factors are presented for their associations with prevalence (per The Prevalence Interval for Microbiome Evaluation, PIME analysis) or the total abundance or relative differential abundance (DESeq2) of key genera. Genera were selected based on Kruskal-Wallis or Mann-Whitney U tests with an adjusted p value for false discovery rate.

^A^Binned 1-3 months, 4-7 months, or 8-9 months.

^B^1-2 meals per week, 3-5 meals per week, Daily, or Seldom.

^C^During Pregnancy; Yes or No.

^D^During Infancy; 1-2 times, 3-5 times, or Never.

Of the significant core microbes, 66% (n=6) were correlated to the presence or absence of HLA haplotypes; however, only 29% (n=2) of the bacteria identified in the differential expression analysis were associated with HLA. *Monoglobus*, found in both analyses, was impacted by both DR13-DQ603 and DR3-DQ2.5. *Butyricicoccus* and *Collinsella* were also correlated with DR13-DQ603. DR15-DQ602 was associated with *Butyricicoccus* as well as *Lachnospiraceae NK4A136 group*. *Erysipelatoclostridium* was correlated with DR14-DQ5, whereas *Terrisporobacter* was specifically correlated with DR14-DQ503. *Barnesiella*, the only non-core microbe impacted by HLA, was associated with DR7-DQ2.2 (**Figures S3**, **S4**).

Dietary factors (such as duration of total or exclusive breastfeeding and weekly meals involving eggs or beef) are associated with one (11%) of the core microbes, *Collinsella*, yet four (57%) of the microbes from differential expression analysis, *Anaeroglobus, Eubacterium, Senegalimassilia, and UCG-002*. Gastroenteritis in the first year of life was associated with three core microbes, *Lachnospiraceae NK4A136 group, Roseburia*, and *Ruminococcus*, and none of the microbes identified in the differential abundance analysis. Biological sex was associated with the greatest number of genera, six total, in both the core microbiome analysis (*Lachnospiraceae NK4A136 group* and *Ruminococcus)* and the differential abundance analysis (*Candidatus Soleaferrea*, *Eubacterium*, and *Senegalimassilia*), as well as *Monoglobus* which was significant in both analyses.

DR3-DQ2.5 was present in a higher proportion of fCD infants compared to all controls, 50% vs. 22% (padj < 0.05), but no other significant factors were found for the selected bacteria (**Table S4**).

## Discussion

This research illustrates through the gut microbiome analysis of 1478 one-year-old infants that microbiome differences exist, both in prevalence and abundance, in the infant’s gut up to 19 years prior to celiac disease diagnosis. The average age of diagnosis of the 26 infants with future CD was 10.96 years, ranging between 2 and 20 years old. Despite the considerable time span between the age of diagnosis and stool collection, we observed microbial differences between healthy controls and fCD infants. Prevalence is a metric that is often overlooked in microbiome studies. In this investigation, we considered both differential abundance of the bacteria, as is customary, and prevalence of the bacteria, which is calculated irrespective of abundance. Whether the bacterium is present or absent may be physiologically meaningful, as may be the architecture of the “core” microbiome.

Core microbiomes were determined using genera prevalent in 60% of fCD cases or controls (n=52). The prevalence threshold was repeated with 100 unique case-control pairings to account for inherent differences in the distribution of controls. We defined genera of interest as those that were significant in 50 or more iterations, which provides further confidence that these core microbiome differences persist, despite the inherent variability of the control constituents.

Three core microbes were higher in fCD infants, *Erysipelatoclostridium*, *Haemophilus*, and *Lachnospiraceae NK4A136 group. Erysipelatoclostridium* and *Haemophilus* are opportunistic pathogens correlated with disease (Shao et al., 2017; Aranaz et al., 2021; Barberio et al., 2022; Cao et al., 2021; Liu et al., 2021; Milosavljevic et al., 2021). *Lachnospiraceae NK4A136 group* is a butyrate producer correlated with both health and disease (Ma et al., 2020; Stadlbauer et al., 2020; Park et al., 2022). Since *Lachnospiraceae NK4A136* group is registered as an uncultured bacterium in the SILVA database, more research is needed to determine the true characteristics of this genus.

*Monoglobus*, unique for being the only genus significant in both core and differentially abundant microbes, is understudied but could be a valuable marker for health against CD. *M. pectinilyticus*, a pectin degrader, is the only current species described (Kim et al., 2019). Further research is needed to determine strain-specific consequences of this genus on host gut health.

Of the differentially abundant microbes, *Candidatus Soleaferrea* stands out for being absent in fCD infants but present in approximately 20% of the control cohorts. There is evidence that *Candidatus Soleaferrea*, which was absent in fCD infants, has anti-inflammatory properties, but this genus has also been correlated with several autoimmune diseases including CD (Cai et al., 2020; Cao et al., 2021; Zou et al., 2022). Within each genus, species are diverse and have a range of metabolic features, which can have various consequences on host physiology. Metagenomic sequencing and culture methods are necessary to understand the role of these specific microbes in CD etiology.

Most CD gut microbiome studies focus on high-genetic-risk patients or are conducted with subjects who have already received a CD diagnosis (Verdu and Schuppan, 2021). The focus on high-risk patients may obscure the microbial differences since HLA has been shown to influence the gut microbiome diversity (Russell et al., 2019). Here, we benefit from a prospective general-population cohort that affords a diverse distribution of HLA genetic features.

HLA polymorphisms were associated with the core microbiome composition of ABIS infants. Six of the nine genera (66%) were correlated with the presence or absence of HLA haplotypes: DR13-DQ603, DR14-DQ5[03], DR15-DQ602, DR3-DQ2.5. Interestingly, the correlative HLA are not all associated with risk for or protection from CD. Through differential analysis, *Monoglobus* and *Barnesiella* were the only two bacteria with HLA-associated abundance differences. A higher abundance of *Barnesiella* correlated with the absence of HLA-DR7-DQ2.2, a known risk allele for CD. The molecular mimicry theory suggests that similarities between microorganism antigens resemble self-antigens or cross-react with HLA molecules, which may help explain the mechanism behind HLA’s impact on bacterial abundance (Ogasawara et al., 1986). Core microbes could be impacted by this molecular mimicry mechanism, while differentially abundant bacteria in fCD appeared to be more associated with external environmental factors rather than HLA.

This study was limited by the number of infants who later developed CD due to the nature of the general population study. The average rate of CD in Sweden is approximately 2­–3%, with regional differences (Myléus et al., 2009). In the current study, the CD diagnosis incidence is 1.8%. To account for the imbalance of fCD cases and controls, we use a novel algorithm that utilizes iterations of randomized matching to create 100 unique case-control cohorts, then used the genera present in at least half of these iterations.

Altogether, these data illustrate the need to consider a broader range of HLA haplotypes in investigating the role of the gut microbiome in CD, as both genetic and environmental factors bear consequence on prevalence and differential abundance of gut microbes. Without looking into non-risk HLA genotypes in CD studies, significant differences in the gut microbiome could be obscured based on the distribution of HLA genetics in the cohort.

## Data availability statement

The forward and reverse 16S raw sequencing data generated in this study is available through the NCBI Sequence Read Archive under BioProject PRJNA854900.HLA genotypes and associated sample metadata used for statistical comparison are available in the source data file. R codes used for statistical comparisons and figure generation are available at: https://github.com/PMilletich/Celiac_Disease_Pipeline.

## Author contributions

TM, AA, JT, and JRT performed data analysis. JT performed the laboratory analysis. TM, MRM, JonL, DA, EWT, and JohL did the writing. JohL developed the ABI cohort. ET and JohL conceived of the project. All authors contributed to the article and approved the submitted version.

## Funding

ABIS was supported by Barndiabetesfonden (Swedish Child Diabetes Foundation); Swedish Council for Working Life and Social Research, Grant/Award Numbers: FAS2004-1775, FAS2004–1775; Swedish Research Council, Grant/Award Numbers: K2005-72 × -11242-11A and K2008- 69 × -20826-01-4, K2008-69 × -20826-01-4; Östgöta Brandstodsbolag; Medical Research Council of Southeast Sweden (FORSS); JDRF Wallenberg Foundation, Grant/Award Number: K 98-99D-12813-01A; ALF-and LfoU grants from Region Östergötland and Linköping University, Sweden; Joanna Cocozza Foundation.

## Conflict of interest

The authors declare that the research was conducted in the absence of any commercial or financial relationships that could be construed as a potential conflict of interest.

## Publisher’s Note

All claims expressed in this article are solely those of the authors and do not necessarily represent those of their affiliated organizations, or those of the publisher, the editors and the reviewers. Any product that may be evaluated in this article, or claim that may be made by its manufacturer, is not guaranteed or endorsed by the publisher.
