# Supplementary material for: Gut microbiome markers in subgroups of HLA class II genotyped infants signal future celiac disease in the general population: ABIS study
Source: Front Cell Infect Microbiol. 2022 Jul 25;12:920735. doi: 10.3389/fcimb.2022.920735 (PMC9357981; doi:10.3389/fcimb.2022.920735)
Supplement: Supplementary file 1 [file DataSheet_1.pdf]

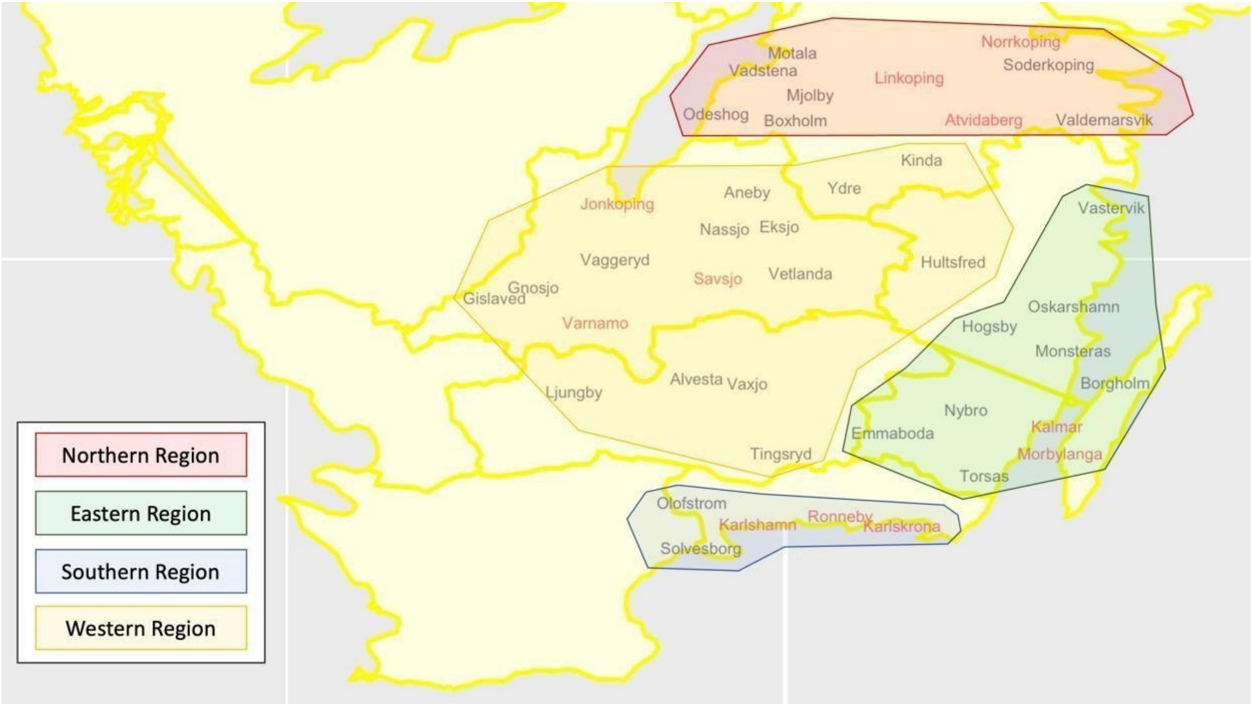

Supplemental Figure 1: Municipalities of ABIS infants at one year of age, binned into regions. Red text representing the municipalities with infants that later become diagnosed with celiac disease.
