# Supplementary material for: Gut microbiome markers in subgroups of HLA class II genotyped infants signal future celiac disease in the general population: ABIS study
Source: Front Cell Infect Microbiol. 2022 Jul 25;12:920735. doi: 10.3389/fcimb.2022.920735 (PMC9357981; doi:10.3389/fcimb.2022.920735)
Supplement: Supplementary file 3 [file DataSheet_3.pdf]

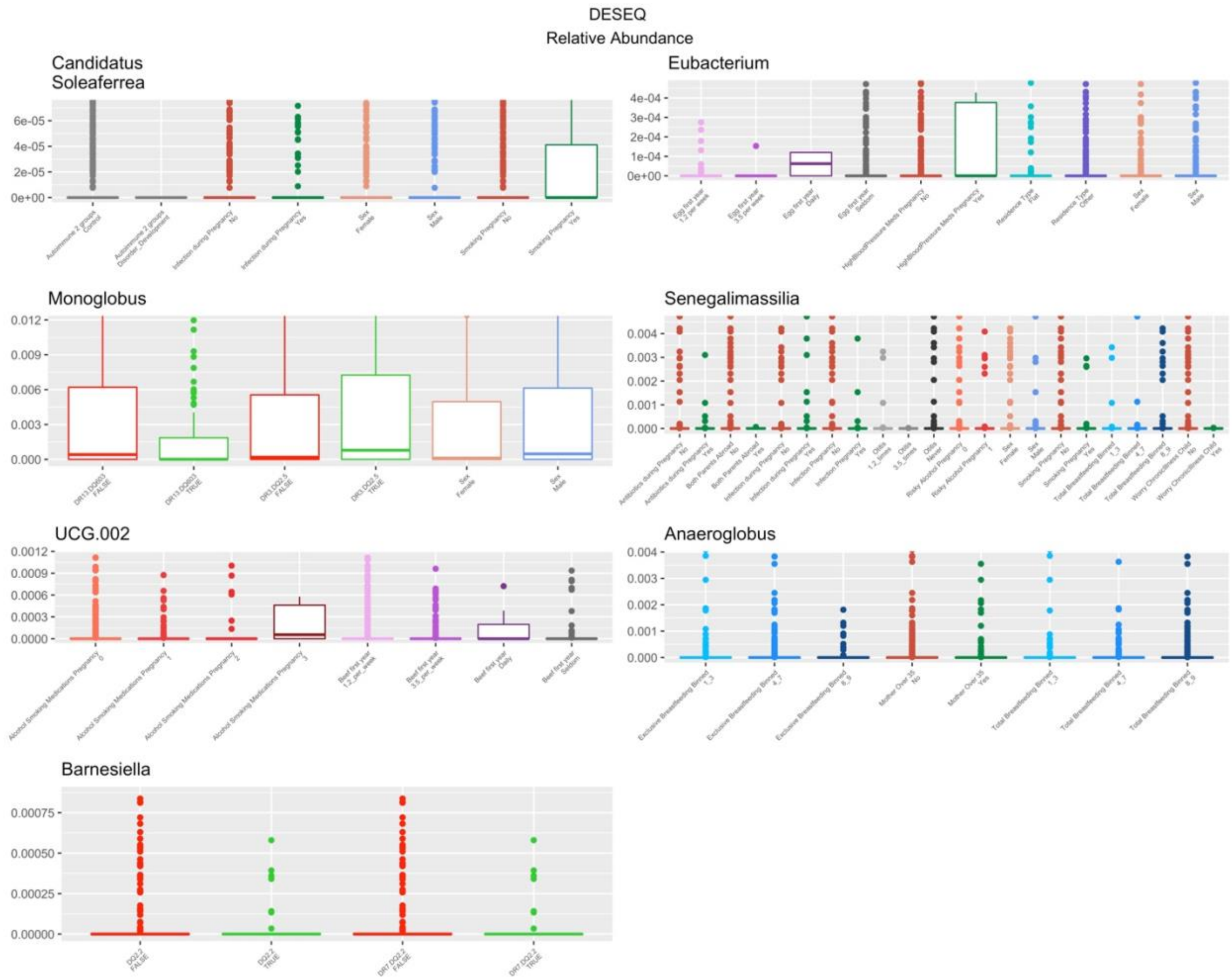

Figure S3: Environmental and genetic factors associated with relative abundance. Genera that were significantly differentially abundant in infants with future celiac disease (fCD) or controls are presented, as identified by binomial distribution expression analysis, DESeq2. Factors were filtered to include those that were significant (padj ≤ 0.05) in both relative abundance and reads/g using a Kruskal-Wallis or Mann-Whitney U test, with a p-value adjusted for false discovery rate.
