# Supplementary material for: Gut microbiome markers in subgroups of HLA class II genotyped infants signal future celiac disease in the general population: ABIS study
Source: Front Cell Infect Microbiol. 2022 Jul 25;12:920735. doi: 10.3389/fcimb.2022.920735 (PMC9357981; doi:10.3389/fcimb.2022.920735)
Supplement: Supplementary file 4 [file DataSheet_4.pdf]

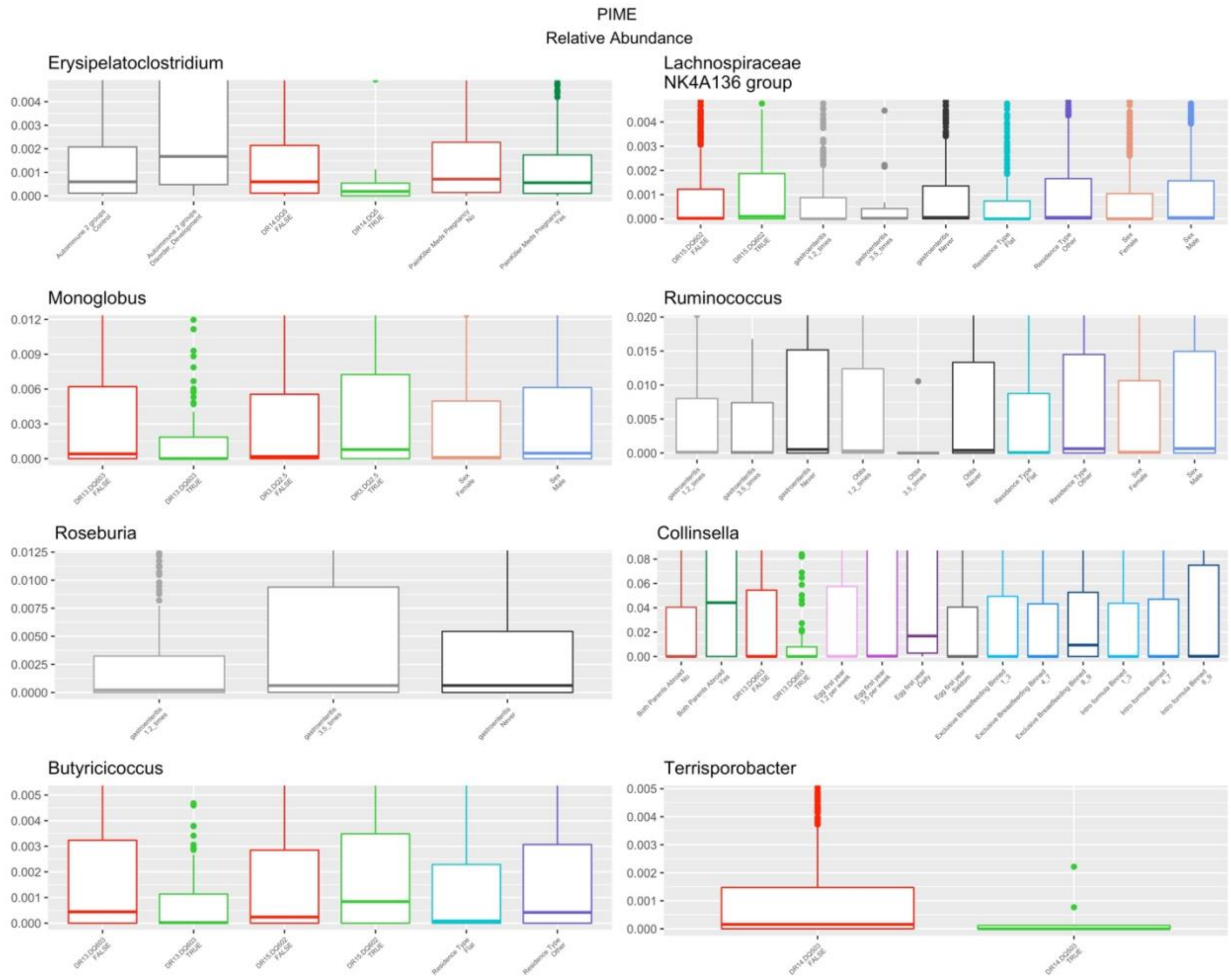

Figure S4: Environmental and genetic factors associated with the relative abundance of core genera with differential prevalence in infants with future celiac disease (fCD). Genera that were identified using The Prevalence Interval for Microbiome Evaluation (PIME) R package are presented. Factors were filtered to include those that were significantly (padj ≤ 0.05) associated with both relative abundance and reads/g by a Kruskal-Wallis or Mann-Whitney U test with false discovery rate correction. The relative abundance is presented on the y axis in each panel.
