# Supplementary material for: Gut microbiome markers in subgroups of HLA class II genotyped infants signal future celiac disease in the general population: ABIS study
Source: Front Cell Infect Microbiol. 2022 Jul 25;12:920735. doi: 10.3389/fcimb.2022.920735 (PMC9357981; doi:10.3389/fcimb.2022.920735)
Supplement: Supplementary file 5 [file DataSheet_5.pdf]

Supplemental Table 1: Environmental, genetic, immunological, or dietary factors and their associations with binomial beta diversity across all infants (n=1478). Four regions of Sweden were defined: Northern, Southern, Eastern, and Western. HLA genetics are presented by the presence of the haplotype, irrespective of dosage. The duration of breastfeeding was binned by 1 to 3 months, 4 to 7 months, and 8-9 months, as was the month of gluten or cow's milk introduction. Weekly meals were grouped as: daily, 1-2 times weekly, 3-5 times weekly, or seldom. Infections during infancy (i.e., gastroenteritis, cold or upper respiratory tract infection, otitis, pneumonia, other infection) were defined as 1-2, 3-5, or never, and were self-reported by the parent in the first-year diary. All other factors were binary classifications.
DF: Degrees of Freedom. SS: Sum of Squares. R2: coefficient of determination. F: F-statistics. Padj: pvalues adjusted for false-discovery-rate.

**Environmental Factors**								
**Variable**	**DF**	**SS**	**R2**	**F**	**Pvalue**		**padj**	
Region	3	8687.9	0.008	4.143	0.001	***	0.011	*
Siblings at birth	1	4503.6	0.005	6.422	0.001	***	0.011	*
Reside in an apartment or flat	1	1756.3	0.002	2.5	0.003	**	0.022	*
Biological Sex	1	1349.4	0.001	1.919	0.015	*	0.083	.
Smoking of the mother during Pregnancy	1	1295.5	0.001	1.84	0.019	*	0.084	.
Both Parents Abroad	1	1234.6	0.001	1.757	0.036	*	0.132	
Mode of Delivery	1	1108.6	0.001	1.579	0.048	*	0.151	
Stressful Life Event during pregnancy	1	979.4	0.001	1.392	0.088	.	0.242	
Father Unemployed	1	926.3	0.001	1.317	0.142		0.347	
Alcohol during Pregnancy	1	825.1	0.001	1.18	0.198		0.436	
Risk events during Pregnancy	1	812.1	0.001	1.17	0.239		0.469	
Worry for Chronic Illness of Child during pregnancy	1	789.1	0.001	1.125	0.271		0.469	
Single Mother	1	786.4	0.001	1.118	0.277		0.469	
Mother with Only Elementary Education	1	757.1	0.001	1.076	0.315		0.483	
Risky Alcohol use during Pregnancy	1	741.9	0.001	1.061	0.341		0.483	
Mother had feelings of Not being Safe during Pregnancy	1	728	0.001	1.034	0.352		0.483	
Father Over 40 years of age	1	733.1	0.001	1.043	0.385		0.483	
Autoimmune status	1	714.2	0.001	1.014	0.401		0.483	
Father with Only Elementary Education	1	702	0.001	0.998	0.417		0.483	
Mother Over 35 years of age	1	672.3	0.001	0.956	0.481		0.529	
Mother with No Support during Pregnancy	1	589.4	0.001	0.837	0.641		0.672	
Mother Unemployed	1	591.2	0.001	0.84	0.682		0.682	
**Human Leukocyte Antigen (HLA) Genetics**								

Variable	DF	SS	R2	F	Pvalue		padj	
DR13-DQ603	1	1462.8	0.002	2.073	0.013	*	0.176	
DR14-DQ5	1	1334.4	0.002	1.891	0.024	*	0.176	
DR15-DQ602	1	1301.9	0.001	1.845	0.024	*	0.176	
DR4-DQ7	1	1058.9	0.001	1.5	0.058	.	0.319	
DR3-DQ2.5	1	934.2	0.001	1.323	0.136		0.479	
DR7-DQ9	1	923.5	0.001	1.308	0.156		0.479	
DR14-DQ503	1	892.5	0.001	1.264	0.175		0.479	
DR15-DQ601	1	867.7	0.001	1.229	0.196		0.479	
DQ2.5	1	835.4	0.001	1.183	0.196		0.479	
DR9-DQ9	1	679.2	0.001	0.962	0.472		0.916	
DR13-DQ604	1	663.4	0.001	0.939	0.515		0.916	
DR16-DQ502	1	646	0.001	0.915	0.544		0.916	
DR7-DQ2.5	1	595.2	0.001	0.843	0.654		0.916	
DR16-DQ5	1	565	0.001	0.8	0.694		0.916	
DQ2.2	1	556.3	0.001	0.788	0.749		0.916	
DR7-DQ2.2	1	556.3	0.001	0.788	0.750		0.916	
DR5-DQ7	1	540.1	0.001	0.765	0.780		0.916	
DR4-DQ8	1	526.7	0.001	0.746	0.810		0.916	
DQ8	1	526.7	0.001	0.746	0.813		0.916	
DR7-DQ2	1	519.4	0.001	0.735	0.835		0.916	
DR8-DQ4	1	486.4	0.001	0.689	0.874		0.916	
DR1-DQ5	1	348.7	0	0.494	0.996		0.996	
**Dietary Factors**								
**Variable**	**DF**	**SS**	**R2**	**F**	**Pvalue**		**padj**	
Duration (mo) of Total Breastfeeding	2	2315.7	0.003	1.631	0.014	*	0.112	
Duration (mo) of Exclusive Breastfeeding	2	2067.7	0.002	1.458	0.036	*	0.131	
Weekly meals with Beef during Infancy	3	2894.5	0.003	1.359	0.049	*	0.131	
Month at which Formula was Introduced	2	1570.7	0.002	1.099	0.298		0.596	
Weekly meals with Egg during Infancy	3	2136	0.002	1.001	0.405		0.648	
Weekly meals with Pork during Infancy	3	2019.5	0.002	0.948	0.540		0.720	
Month of Gluten Introduction	2	1183.7	0.001	0.832	0.750		0.837	
Month of Cow's milk Introduction	2	1117.3	0.001	0.786	0.837		0.837	
**Immunological Factors**								
**Variable**	**DF**	**SS**	**R2**	**F**	**Pvalue**		**padj**	
High Blood Pressure Medication during Pregnancy	1	1189	0.001	1.701	0.037	*	0.620	
Infection with antibiotics during Infancy	3	2748.2	0.003	1.289	0.079	.	0.620	

Gastroenteritis during Infancy	2	1757.4	0.002	1.234	0.122		0.620	
Infection during Pregnancy	1	893.6	0.001	1.264	0.157		0.620	
Cold or Upper Resp. Tract infection during Infancy	2	1617	0.002	1.137	0.241		0.620	
Antibiotics during Pregnancy	1	771.2	0.001	1.102	0.269		0.620	
Corticosteroids during Pregnancy	1	762.9	0.001	1.094	0.304		0.620	
Total Medication during Pregnancy	1	750.8	0.001	1.078	0.310		0.620	
Otitis during Infancy	2	1403.9	0.002	0.989	0.477		0.771	
Psychiatric Medication during Pregnancy	1	654.6	0.001	0.938	0.515		0.771	
Other Disease during Infancy	2	1361.8	0.002	0.952	0.550		0.771	
Other Medication during Pregnancy	1	624.8	0.001	0.896	0.557		0.771	
Pneumonia during Infancy	2	1267.1	0.002	0.887	0.644		0.781	
Pain Killer Medication during Pregnancy	1	598.9	0.001	0.855	0.651		0.781	
Other Infection during Infancy	2	1219.9	0.002	0.856	0.726		0.817	
Infection as newborn	1	489.6	0.001	0.689	0.876		0.928	
Hormone Preparate during Pregnancy	1	335	0	0.479	0.990		0.990	
