# Supplementary material for: Gut microbiome markers in subgroups of HLA class II genotyped infants signal future celiac disease in the general population: ABIS study
Source: Front Cell Infect Microbiol. 2022 Jul 25;12:920735. doi: 10.3389/fcimb.2022.920735 (PMC9357981; doi:10.3389/fcimb.2022.920735)
Supplement: Supplementary file 6 [file DataSheet_6.pdf]

**Supplemental Table 2**: The Prevalence Interval for Microbiome Evaluation (PIME) analysis summary statistics for the 100 case-control iterations. Statistics for each of the first ten iterations are provided. These ten iterations served as the standard for comparison of the other ninety iterations. In each iteration, an independent set of controls was selected for each infant with future Celiac Disease (fCD) after matching on confounders of the microbiome composition in the ABIS cohort (i.e., geography and presence of siblings at birth). The average of the ten iterations is also provided. The statistics represent the OOB, number of genera retained, and number of sequences at each of the 5% prevalence intervals within each model. The prevalence interval of 60% was selected for subsequent analysis, based on the optimal parameters for balancing a low OOB error with a maximum number of genera and sequences. OOB: Out of Bag Error rate. Genera: Number of genera retained. NSeqs: Number of sequences												
	Average of 10 iterations			Iteration 1			Iteration 2			Iteration 3		
Interval	OOB	Genera	Nseqs	OOB	Genera	Nseqs	OOB	Genera	Nseqs	OOB	Gener	Nseqs
5	38.3	141.4	5.7E+09	41.03	140	8.5E+09	41.03	138	4.5E+09	41.03	144	4.7E+09
10	34.1	115.2	5.6E+09	37.18	116	8.5E+09	35.9	115	4.4E+09	34.62	116	4.7E+09
15	32.7	101.8	5.6E+09	35.9	104	8.5E+09	34.62	104	4.4E+09	33.33	106	4.6E+09
20	29.7	78.4	5.6E+09	33.33	77	8.4E+09	28.21	82	4.4E+09	30.77	80	4.6E+09
25	27.6	68.7	5.6E+09	29.49	68	8.4E+09	16.67	69	4.4E+09	32.05	70	4.5E+09
30	22.4	63.2	5.6E+09	30.77	61	8.4E+09	12.82	62	4.4E+09	21.79	66	4.5E+09
35	11.3	58.9	5.5E+09	7.69	58	8.3E+09	11.54	58	4.3E+09	8.97	59	4.5E+09
40	7.3	55.8	5.5E+09	7.69	55	8.3E+09	8.97	56	4.2E+09	6.41	56	4.5E+09
45	5.5	52.1	5.4E+09	7.69	51	8.2E+09	5.13	52	4.2E+09	7.69	52	4.4E+09
50	6.4	44.1	5.3E+09	7.69	44	8.1E+09	8.97	43	4.1E+09	2.56	46	4.3E+09
55	3.8	40.8	5.2E+09	2.56	40	7.9E+09	6.41	39	4.0E+09	6.41	42	4.2E+09
60	2.8	35.3	5.0E+09	3.85	34	7.8E+09	3.85	35	3.8E+09	1.28	37	4.2E+09
65	2.7	32.3	4.9E+09	6.41	30	7.5E+09	1.28	32	3.7E+09	0	34	4.1E+09
70	1.9	27.2	4.6E+09	3.85	25	7.3E+09	2.56	26	3.5E+09	1.28	29	3.8E+09
75	5.9	22.2	4.4E+09	3.85	22	7.2E+09	2.56	22	3.4E+09	3.85	24	3.5E+09
80	3.8	20.6	4.3E+09	3.85	22	7.2E+09	3.85	20	3.4E+09	1.28	23	3.5E+09
85	1.3	16	4.0E+09	1.28	15	6.6E+09	5.13	14	2.8E+09	0	15	2.9E+09
90	0.9	13.8	3.8E+09	1.28	13	6.6E+09	2.56	13	2.8E+09	0	14	2.9E+09
95	0.4	10.2	3.5E+09	1.28	10	6.4E+09	0	10	2.6E+09	1.28	10	2.7E+09

Interval	Iteration 4			Iteration 5			Iteration 6			Iteration 7		
	OOB	Genera	Nseqs	OOB	Genera	Nseqs	OOB	Genera	Nseqs	OOB	Genera	Nseqs
5	34.62	142	8.2E+09	39.74	141	4.6E+09	33.33	140	5.5E+09	34.62	142	6.0E+09
10	33.33	113	8.2E+09	29.49	116	4.6E+09	32.05	110	5.5E+09	33.33	118	6.0E+09
15	29.49	101	8.2E+09	30.77	100	4.5E+09	25.64	98	5.5E+09	38.46	106	5.9E+09
20	26.92	73	8.2E+09	24.36	80	4.5E+09	30.77	76	5.5E+09	25.64	85	5.9E+09
25	34.62	66	8.1E+09	26.92	73	4.5E+09	14.1	67	5.4E+09	29.49	72	5.9E+09
30	15.38	61	8.1E+09	12.82	64	4.5E+09	16.67	62	5.4E+09	32.05	67	5.8E+09
35	10.26	60	8.1E+09	8.97	59	4.4E+09	11.54	58	5.4E+09	16.67	61	5.8E+09
40	10.26	56	8.1E+09	7.69	55	4.4E+09	7.69	54	5.3E+09	5.13	57	5.7E+09
45	3.85	52	8.0E+09	3.85	53	4.4E+09	5.13	50	5.3E+09	5.13	54	5.6E+09
50	3.85	45	7.9E+09	3.85	46	4.1E+09	8.97	42	5.2E+09	3.85	44	5.2E+09
55	1.28	43	7.8E+09	2.56	43	4.0E+09	2.56	40	5.1E+09	3.85	40	5.1E+09
60	6.41	35	7.4E+09	5.13	36	3.9E+09	1.28	34	4.9E+09	1.28	36	5.0E+09
65	10.26	32	7.4E+09	2.56	33	3.8E+09	1.28	31	4.8E+09	1.28	33	4.8E+09
70	1.28	27	7.0E+09	0	30	3.5E+09	2.56	27	4.7E+09	1.28	27	4.5E+09
75	3.85	21	6.7E+09	2.56	25	3.4E+09	1.28	21	4.2E+09	8.97	22	4.4E+09
80	2.56	21	6.7E+09	3.85	21	3.4E+09	3.85	19	4.2E+09	5.13	20	4.3E+09
85	1.28	17	6.4E+09	0	18	3.1E+09	1.28	16	3.8E+09	2.56	16	4.0E+09
90	2.56	14	6.2E+09	1.28	14	2.9E+09	0	15	3.7E+09	0	13	3.7E+09
95	0	10	5.8E+09	1.28	11	2.8E+09	0	10	2.7E+09	0	10	2.8E+09

Interval	Iteration 8			Iteration 9			Iteration 10		
	OOB	Genera	Nseqs	OOB	Genera	Nseqs	OOB	Genera	Nseqs
5	38.46	146	5.2E+09	39.74	140	5.5E+09	39.74	141	4.0E+09
10	35.9	114	5.2E+09	34.62	117	5.5E+09	34.62	117	3.9E+09
15	30.77	99	5.2E+09	34.62	100	5.4E+09	33.33	100	3.9E+09
20	34.62	77	5.1E+09	34.62	75	5.4E+09	28.21	79	3.9E+09
25	32.05	69	5.1E+09	35.9	67	5.4E+09	24.36	66	3.9E+09
30	32.05	63	5.1E+09	33.33	65	5.4E+09	16.67	61	3.9E+09
35	10.26	60	5.1E+09	19.23	58	5.3E+09	7.69	58	3.8E+09
40	6.41	57	5.1E+09	6.41	56	5.3E+09	6.41	56	3.8E+09
45	3.85	53	5.0E+09	5.13	52	5.2E+09	7.69	52	3.8E+09
50	5.13	44	4.9E+09	10.26	44	5.2E+09	8.97	43	3.7E+09
55	3.85	40	4.8E+09	5.13	41	5.0E+09	3.85	40	3.6E+09
60	1.28	38	4.7E+09	2.56	34	4.9E+09	1.28	34	3.5E+09
65	0	35	4.6E+09	1.28	32	4.8E+09	2.56	31	3.5E+09
70	1.28	31	4.3E+09	2.56	25	4.2E+09	2.56	25	3.1E+09
75	2.56	22	4.1E+09	23.08	21	4.0E+09	6.41	22	3.0E+09
80	3.85	20	4.1E+09	8.97	19	3.9E+09	1.28	21	2.9E+09
85	0	18	3.9E+09	1.28	16	3.6E+09	0	15	2.6E+09
90	0	14	3.5E+09	1.28	15	3.6E+09	0	13	2.6E+09
95	0	11	3.4E+09	0	10	3.2E+09	0	10	2.5E+09
