# Supplementary material for: Gut microbiome markers in subgroups of HLA class II genotyped infants signal future celiac disease in the general population: ABIS study
Source: Front Cell Infect Microbiol. 2022 Jul 25;12:920735. doi: 10.3389/fcimb.2022.920735 (PMC9357981; doi:10.3389/fcimb.2022.920735)
Supplement: Supplementary file 7 [file DataSheet_7.pdf]

Supplemental Table 3: Environmental, genetic, immunological, and dietary factors associated with total (reads/g) abundance and relative abundance across all infants (n=1478). Only the genera that were differentially abundant or prevalent in fCD, per DESeq2 or PIME analyses respectively, were tested. HLA genetics are presented by the presence of the haplotype, irrespective of dosage. Duration of breastfeeding was binned by 1-3, 4-7, or 8-9 months, as was the month of gluten, formula, or cow's milk introduction. Weekly meals were grouped as: daily, 1-2 times weekly, 3-5 times weekly, or seldom. Infections during infancy (i.e., gastroenteritis, cold or upper respiratory tract infection, otitis, pneumonia, other infection) were defined as: 1-2, 3-5, or never and were self-reported by the parent in the first-year diary. All other factors were binary classifications. The p values (padj) were adjusted for false-discovery-rate.

	Genus	Variable	Reads/g		Relative Abundance	
			Pvalue	padj	Pvalue	padj
Bacteria with differential prevalence in fCD	*Butyricicoccus*	Reside in an apartment or flat	7.41E-5	6.67E-4	8.86E-4	3.99E-3
		DR13-DQ603	1.17E-3	1.05E-2	8.33E-5	7.50E-4
		DR15-DQ602	8.84E-3	6.89E-2	8.83E-3	7.95E-2
	Collinsella	Both Parents Abroad	2.57E-2	2.31E-1	2.58E-3	2.32E-2
		DR13-DQ603	3.96E-2	1.19E-1	1.74E-2	5.23E-2
		Egg consumption during first year	1.87E-3	1.68E-2	5.16E-3	4.65E-2
		Duration of Exclusive Breastfeeding (months)	1.42E-2	1.28E-1	1.97E-2	1.63E-1
		Month of Formula Introduction	1.89E-2	1.70E-1	3.27E-2	2.94E-1
	Erysipelato-clostridium	DR14-DQ5	1.74E-2	1.56E-1	1.56E-2	1.40E-1
		Pain Killer Medications during Pregnancy	2.29E-2	2.06E-1	1.84E-2	1.65E-1
	Lachnospiraceae NK4A136 group	Reside in an apartment or flat	1.54E-4	6.94E-4	4.63E-4	3.99E-3
		DR15-DQ602	1.53E-2	6.89E-2	2.03E-2	9.13E-2
		Gastroenteritis during first year	4.22E-2	1.33E-1	2.81E-2	1.26E-1
		Biological Sex	3.13E-2	1.02E-1	3.62E-2	8.06E-2
	Roseburia	Gastroenteritis during first year	4.01E-2	1.33E-1	4.81E-2	1.31E-1
	Ruminococcus	Reside in an apartment or flat	3.79E-3	1.14E-2	1.23E-2	3.70E-2
		Gastroenteritis during first year	4.42E-2	1.33E-1	2.48E-2	1.26E-1
		Otitis during first year	2.17E-2	1.95E-1	2.12E-2	1.90E-1
		Biological Sex	4.03E-2	1.02E-1	2.40E-2	7.79E-2
	Terrisporobacter	DR14-DQ503	1.96E-2	1.77E-1	3.62E-2	3.25E-1

Bacteria with differential abundance in fCD	*Anaeroglobus*	Duration of Exclusive Breastfeeding (months)	4.53E-3	3.17E-2	5.12E-3	3.58E-2
		Mother Over 35 years of age	2.40E-3	1.68E-2	3.59E-3	2.51E-2
		Duration of Total Breastfeeding (months)	4.27E-2	1.49E-1	3.74E-2	1.31E-1
	Barnesiella	DR7-DQ2.2	4.14E-2	2.89E-1	4.96E-2	3.47E-1
	Candidatus Soleaferrea	Autoimmune status	1.34E-2	9.37E-2	1.34E-2	9.37E-2
		Infection during Pregnancy	3.20E-2	1.12E-1	3.24E-2	9.90E-2
		Biological Sex	4.53E-2	7.92E-2	3.36E-2	7.84E-2
		Smoking of the mother during Pregnancy	1.94E-3	1.36E-2	8.66E-4	6.06E-3
	Eubacterium	Reside in an apartment or flat	1.41E-2	9.15E-2	1.77E-2	1.24E-1
		Egg consumption during first year	6.11E-3	4.28E-2	8.20E-3	5.74E-2
		High Blood Pressure Medications during Pregnancy	4.44E-4	3.11E-3	5.13E-4	3.59E-3
		Biological Sex	4.35E-2	7.92E-2	4.85E-2	8.48E-2
	Senegalimassilia	Antibiotics during Pregnancy	3.00E-2	2.10E-1	3.34E-2	2.34E-1
		Both Parents Abroad	1.12E-2	7.83E-2	1.01E-2	7.10E-2
		Infection during Pregnancy	1.17E-2	8.17E-2	1.09E-2	7.65E-2
		Otitis during first year	2.93E-2	2.05E-1	2.58E-2	1.80E-1
		Risky Alcohol use during Pregnancy	2.50E-2	1.75E-1	2.92E-2	1.98E-1
		Biological Sex	1.74E-3	1.22E-2	1.53E-3	1.07E-2
		Smoking during Pregnancy	1.40E-2	4.89E-2	1.46E-2	5.12E-2
		Duration of Total months of Breastfeeding	4.10E-2	1.49E-1	3.25E-2	1.31E-1
		Worry for Chronic Illness of the Child during pregnancy	3.30E-3	2.31E-2	2.77E-3	1.94E-2
	UCG.002	Alcohol use, Smoking, and Medications of the mother during Pregnancy	4.75E-2	3.33E-1	3.06E-2	2.14E-1

		Beef during the first year	5.00E-2	2.55E-1	2.79E-2	1.96E-1
Both	*Monoglobus*	DR13-DQ603	3.97E-3	1.79E-2	1.45E-3	6.51E-3
		DR3-DQ2.5	2.69E-2	2.42E-1	2.84E-2	2.55E-1
		Biological Sex	2.10E-2	1.02E-1	2.60E-2	7.79E-2
