# Supplementary material for: Gut microbiome markers in subgroups of HLA class II genotyped infants signal future celiac disease in the general population: ABIS study
Source: Front Cell Infect Microbiol. 2022 Jul 25;12:920735. doi: 10.3389/fcimb.2022.920735 (PMC9357981; doi:10.3389/fcimb.2022.920735)
Supplement: Supplementary file 8 [file DataSheet_8.pdf]

Supplemental Table 4. Environmental and genetic factors associated with future celiac diagnosis (fCD). Results are based on chi-squared tests comparing fCD (n=26) with all controls (n=1452). Samples with missing data were removed from the calculations at each comparison. HLA genetics are presented by the presence of the haplotype, irrespective of dosage. Duration of breastfeeding was binned by 1-3, 4-7, or 8-9 months, as was the month of gluten or cow's milk introduction. Weekly meals were grouped as: daily, 1-2 times weekly, 3-5 times weekly, or seldom. Infections during infancy (i.e., gastroenteritis, cold or upper respiratory tract infection, otitis, pneumonia, other infection) were defined as: 1-2, 3-5, or never, and were self-reported by the parent in the first-year diary. All other factors were binary classifications.

Human Leukocyte Antigen (HLA) Genetics				
**Variable**	**Pvalue**		**FDR**	
DQ2.5	0.002	**	0.049	*
DR3-DQ2.5	0.004	**	0.049	*
DR1-DQ5	0.01	**	0.077	.
DQ8	0.18		0.726	
DR4-DQ8	0.192		0.726	
DR7-DQ2.5	0.198		0.726	
DR7-DQ9	0.247		0.776	
DR13-DQ603	0.347		0.954	
DR4-DQ7	0.489		1	
DR7-DQ2	0.516		1	
DR5-DQ7	0.579		1	
DR8-DQ4	0.714		1	
DQ2.2	0.719		1	
DR7-DQ2.2	0.726		1	
DR13-DQ604	1		1	
DR14-DQ5	1		1	
DR14-DQ503	1		1	
DR15-DQ601	1		1	
DR15-DQ602	1		1	
DR16-DQ5	1		1	
DR16-DQ502	1		1	
DR9-DQ9	1		1	
**Dietary Factors**				
**Variable**	**Pvalue**		**FDR**	
Duration of Total Breastfeeding (months)	0.023	*	0.134	
Month of Formula Introduction	0.033	*	0.134	
Weekly meals with Pork during first year	0.224		0.598	
Month of Gluten Introduction	0.662		0.984	
Weekly meals with Egg during first year	0.765		0.984	

Weekly meals with Beef during first year	0.551		0.984	
Duration of Exclusive Breastfeeding (months)	0.861		0.984	
Month of Cow's milk Introduction	1		1	
**Immunological Factors**				
**Variable**	**Pvalue**		**FDR**	
Cold or Upper Respiratory Tract Infection during Infancy	0.007	**	0.126	
Infection as newborn	0.103		0.666	
Total Medications During Pregnancy	0.111		0.666	
Otitis during Infancy	0.215		0.675	
Pain Killer Medications during Pregnancy	0.285		0.675	
High Blood Pressure Medications during Pregnancy	0.288		0.675	
Antibiotics during Pregnancy	0.306		0.675	
Other Disease during Infancy	0.364		0.675	
Corticosteroids during Pregnancy	0.401		0.675	
Other Infection during Infancy	0.412		0.675	
Pneumonia during Infancy	0.502		0.753	
Infection with antibiotics during Infancy	0.76		0.924	
Gastroenteritis during Infancy	0.769		0.924	
Other Medications during Pregnancy	0.8		0.924	
Infection during Pregnancy	0.821		0.924	
Psychiatric Medications during Pregnancy	1		1	
Hormone Preparates during Pregnancy	1		1	
**Other Factors**				
**Variable**	**Pvalue**		**FDR**	
Biological Sex	0.101		1	
Mother Unemployed	0.176		1	
Siblings at birth	0.211		1	
Stressful Life Event during pregnancy	0.258		1	
Mother Over 35 years of age	0.35		1	
Region of Sweden	0.415		1	
Smoking of the mother during Pregnancy	0.444		1	
Alcohol use during Pregnancy	0.47		1	
Risky Alcohol Pregnancy	0.716		1	
Reside in an apartment or flat	0.836		1	
Alcohol Smoking Medications Pregnancy	0.904		1	
Mode of Delivery	1		1	
Father with Only Elementary Education	1		1	
Mother with Only Elementary Education	1		1	
Father Unemployed	1		1	
Both Parents Abroad	1		1	
Single Mother	1		1	

Mother with No Support during Pregnancy	1		1	
Mother with feelings of Not being Safe during Pregnancy	1		1	
Worry for Chronic Illness of the Child during pregnancy	1		1	
Father Over 40 years of age	1		1	
