# Supplementary material for: Gut microbiome markers in subgroups of HLA class II genotyped infants signal future celiac disease in the general population: ABIS study
Source: Front Cell Infect Microbiol. 2022 Jul 25;12:920735. doi: 10.3389/fcimb.2022.920735 (PMC9357981; doi:10.3389/fcimb.2022.920735)
Supplement: Supplementary file 2 [file DataSheet_2.pdf]

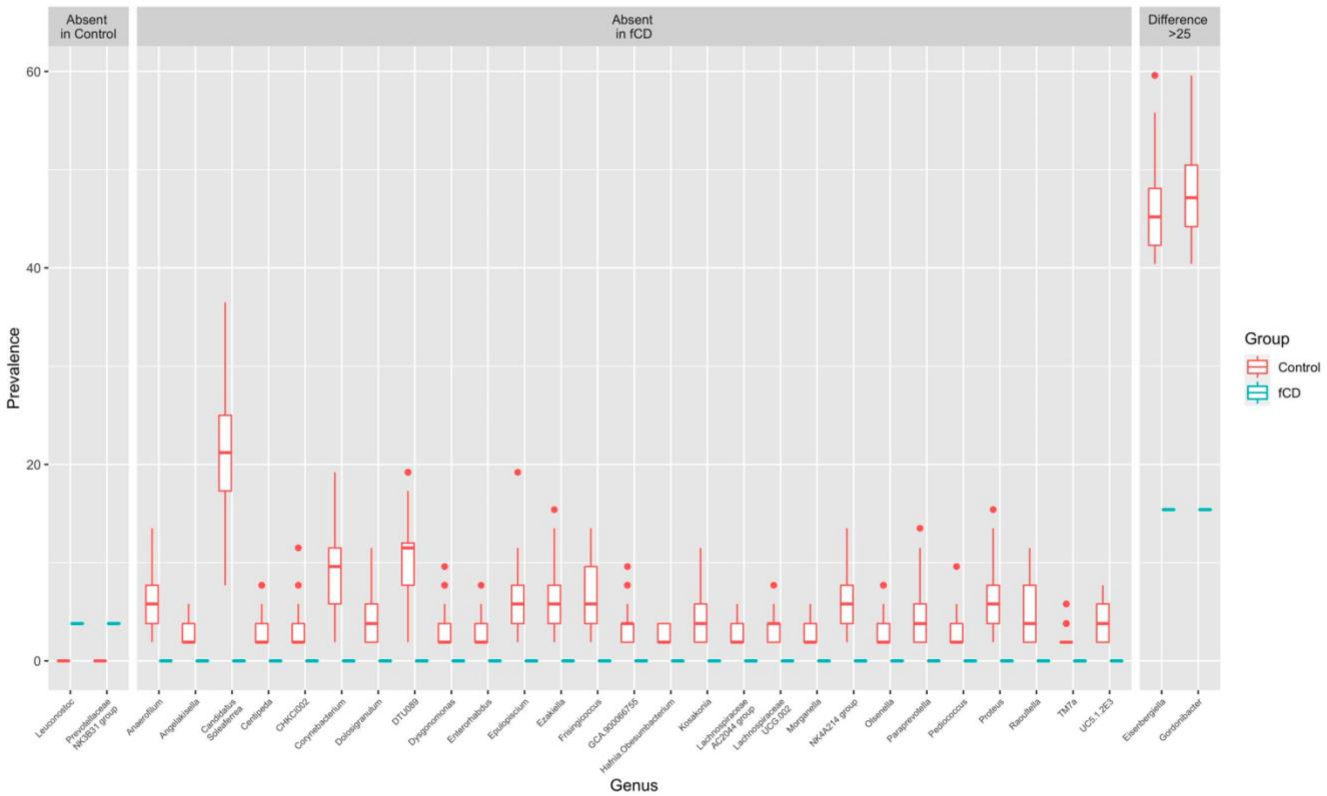

Figure S2: Prevalence differences between infants with future celiac disease (fCD) and Controls. Genera that were, in at least half of the case-control permutations, either absent in controls (n=52), absent in fCD (n=26), or had a difference in prevalence greater than 25%.
